# Patient and Caregiver Perspectives on Hospital-at-Home in Saudi Arabia

**DOI:** 10.1001/jamanetworkopen.2026.3522

**Published:** 2026-03-25

**Authors:** Mahamadu Maida, Ahmed Jameel Al Jameel, Rawan Jassim AlGhawi, Hesa Albanki, Lama Almahrous, Kawthar Al Hussain, Anwar Bader Alotaibi

**Affiliations:** 1 Internal Medicine Department, Johns Hopkins Aramco Healthcare Hospital, Dhahran, Saudi Arabia; 2 Research Department, Johns Hopkins Aramco Healthcare Hospital, Dhahran, Saudi Arabia

## Abstract

This cross-sectional study evalutes patients’ perspectives and examine caregivers’ readiness for hospital-at-home services in Saudi Arabia.

## Introduction

Hospital-at-home (HH) services offer hospital-level treatment in patients’ homes, improving patient satisfaction, lowering costs, and reducing hospital admissions.^1^ Acceptability of HH may be influenced by cultural expectations, caregiving norms, and the availability of home health services, which may differ from more established settings. Despite the provided evidence of HH benefits, its use in the Middle East remains limited.^2^ This study aimed to assess patients’ perspectives and examine caregivers’ readiness for HH services in Saudi Arabia.

## Methods

We conducted a cross-sectional survey of adult patients who received HH health services for conditions such as postsurgical recovery, acute infections, and chronic disease management between April 2024 and December 2024 at Johns Hopkins Aramco Healthcare in Dhahran, Saudi Arabia, through direct telephone interviews. Participants completed an Arabic questionnaire to evaluate their acceptability, perceived effectiveness, safety, and convenience of using the HH model. Additionally, we assessed the caregivers’ willingness to perform common medical tasks such as pain management. HH services consist of daily nursing visits, medication administration, monitoring of vital signs, coordination of laboratory tests, and provision of supportive therapies as indicated. A portion of patients included were determined using OpenEpi Version 3 (Open Source Epidemiologic Statistics for Public Health) based on the reference study.^3^

Patient characteristics were compared using descriptive statistics and Pearson χ^2^ tests; a 2-sided *P* value less than .05 was considered significant. Weighted percentages with 95% CIs were calculated for all responses; all statistical analyses were performed using R Studio version 2023.06.0 (R Project for Statistical Computing). Ethical approval was obtained from Johns Hopkins Aramco Healthcare, and verbal informed consent was obtained. The study followed the Strengthening the Reporting of Observational Studies in Epidemiology (STROBE) reporting guideline for cross-sectional studies.

## Results

Of 292 individuals, 184 responded to the survey (63% response rate). The acceptability of HH care was 88.0% (95% CI, 82.8%-91.8%), neutrality was 2.7% (95% CI, 1.2%-6.2%), and unacceptability was 9.2% (95% CI, 5.8%-14.3%) (Table 1).

**Table 1.** Distribution of Sociodemographic and Health Characteristics by Reported Acceptability of Hospital-at-Home Care

Characteristic	Participants, % (95% CI)				P value
	Total (N = 184)	Acceptability of hospital-at-home (n = 162)	Neutrality of hospital-at-home (n = 5)	Unacceptability of hospital-at-home (n = 17)	
Sex					
Female	53.3 (46.1-60.4)	60.5 (52.8-67.7)	40.0 (11.8-76.9)	52.9 (30.9-73.8)	.56
Male	46.7 (39.6-53.9)	39.5 (32.3-47.2)	60.0 (23.1-88.2)	47.1 (26.2-69.0)	
Age, y					
14-19	0.5 (0.1-3.0)	0.6 (0.1-3.4)	NA	NA	.24
20-34	4.3 (2.1-8.7)	4.3 (2.1-8.6)	NA	5.9 (1.0-26.9)	
35-54	7.6 (4.5-12.6)	8.6 (5.2-14.0)	NA	NA	
55-64	13.0 (8.8-18.8)	10.5 (6.7-16.2)	20.0 (3.6-62.4)	35.3 (17.3-58.7)	
≥65	74.5 (67.5-80.4)	75.9 (68.8-81.9)	80.0 (37.6-96.4)	58.8 (36.0-78.4)	
Nationality					
Non-Saudi	2.2 (0.9-5.5)	2.5 (1.0-6.2)	NA	NA	.76
Saudi	97.8 (94.5-99.1)	97.5 (93.8-99.0)	100.0 (56.6-100.0)	100.0 (81.6-100.0)	
Marital status					
Divorced	2.2 (0.9-5.5)	2.5 (1.0-6.2)	NA	NA	.67
Married	73.9 (66.9-79.9)	74.1 (66.8-80.2)	60.0 (23.1-88.2)	64.7 (41.3-82.7)	
Single	4.9 (2.4-9.7)	4.3 (2.1-8.6)	NA	11.8 (3.3-34.3)	
Widow	18.5 (13.3-25.2)	19.1 (13.8-25.9)	40.0 (11.8-76.9)	23.5 (9.6-47.3)	
Education					
Advanced degree	3.3 (1.4-7.5)	3.1 (1.3-7.0)	NA	5.9 (1.0-26.9)	.60
Bachelor	12.5 (8.3-18.5)	11.7 (7.6-17.6)	40.0 (11.8-76.9)	11.8 (3.3-34.3)	
Diploma	6.0 (3.2-11.0)	5.6 (2.9-10.2)	NA	11.8 (3.3-34.3)	
Elementary	17.9 (12.7-24.7)	16.0 (11.2-22.5)	NA	29.4 (13.3-53.1)	
High school	10.3 (6.5-16.0)	9.9 (6.2-15.4)	20.0 (3.6-62.4)	11.8 (3.3-34.3)	
Intermediate	7.6 (4.5-12.6)	8.0 (4.7-13.2)	NA	5.9 (1.0-26.9)	
Uneducated	42.4 (35.2-50.0)	45.7 (38.2-53.4)	40.0 (11.8-76.9)	23.5 (9.6-47.3)	
Household income (per month), SAR					
<5000	44.0 (36.7-51.6)	45.7 (38.2-53.4)	NA	41.2 (21.6-64.0)	.03
5000-14 000	28.3 (21.8-35.8)	26.5 (20.3-33.8)	60.0 (23.1-88.2)	29.4 (13.3-53.1)	
15 000-29 000	17.9 (12.7-24.7)	17.9 (12.8-24.5)	NA	29.4 (13.3-53.1)	
30 000-44 000	7.1 (4.0-12.2)	6.2 (3.4-11.0)	40.0 (11.8-76.9)	NA	
>450 000	2.7 (1.2-6.0)	3.7 (1.7-7.8)	NA	NA	
Overall health					
Excellent or very good	17.9 (12.7-24.7)	19.1 (13.8-25.9)	20.0 (3.6-62.4)	5.9 (1.0-26.9)	.58
Good	30.4 (23.8-38.0)	31.5 (24.8-39.0)	40.0 (11.8-76.9)	23.5 (9.6-47.3)	
Fair	29.3 (22.8-36.9)	27.2 (20.9-34.5)	40.0 (11.8-76.9)	41.2 (21.6-64.0)	
Poor	22.3 (16.5-29.5)	22.2 (16.5-29.2)	NA	29.4 (13.3-53.1)	
No. of chronic conditions					
None	3.8 (1.9-7.5)	2.5 (1.0-6.2)	NA	17.6 (6.2-41.0)	.01
1	6.5 (3.6-11.5)	6.2 (3.4-11.0)	25.0 (4.6-69.9)	NA	
≥2	89.7 (83.9-93.5)	91.4 (86.0-94.8)	75.0 (30.1-95.4)	82.4 (58.9-93.8)	
Hospitalization since March 2022	74.5 (67.5-80.4)	72.2 (64.9-78.5)	100.0 (56.6-100.0)	88.2 (65.7-96.7)	.15
Telehealth visit in last 12 mo	70.7 (63.5-77.0)	68.5 (61.0-75.2)	100.0 (56.6-100.0)	94.1 (73.0-98.9)	.03

Abbreviations: NA, not applicable; SAR, Saudi Riyal.

The strong agreement for choosing hospital-level care at home was reported by 59.2% respondents (95% CI, 52.0%-66.1%), and 70.1% (95% CI, 63.1%-76.3%) endorsed home-based physical therapy. In addition, 59.2% respondents (95% CI, 52.0%-66.1%) strongly disagreed that clinician visits were intrusive. Caregiving willingness was high, particularly for medication management (96.2%; 95% CI, 92.4%-98.1%) and pain management (94.6%; 95% CI, 90.3%-97.0%), although it was lower for complex tasks like catheter or feeding tube changes (Table 2). Overall patient satisfaction was 93.0% (95% CI, 88.3%-95.8%), indicating strong support for the model.

**Table 2.** Participant Agreement Ratings Across Domains of Hospital-at-Home Care and Caregiving Tasks

Domain and questionnaire item	Participants, % (95% CI)				
	Strongly agree	Agree	Neutral	Disagree	Strongly disagree
Acceptability					
If I had a choice, I would choose to have hospital-level care at home	59.2 (52.0-66.1)	28.8 (22.7-35.7)	2.7 (1.2-6.2)	8.2 (5.0-13.0)	1.1 (0.3-3.9)
Effectiveness					
People recover faster at home than in the hospital	50.0 (42.8-57.2)	25.5 (19.8-32.3)	10.3 (6.7-15.6)	13.0 (8.9-18.7)	1.1 (0.3-3.9)
Medical care at home can be as good as medical care in the hospital	42.4 (35.5-49.6)	21.7 (16.4-28.2)	11.4 (7.6-16.8)	23.4 (17.8-30.0)	1.1 (0.3-3.9)
Safety					
If I had an emergency at home, it would take too long for medical care to arrive^a^	8.2 (5.0-13.0)	21.2 (15.9-27.7)	22.3 (16.9-28.8)	48.4 (41.3-55.5)	NA
At home, I would be less likely to get confused or catch an infection than in the hospital	54.3 (47.1-61.4)	31.0 (24.7-38.0)	7.6 (4.6-12.4)	7.1 (4.2-11.7)	NA
I would feel safe being treated at home	47.8 (40.7-55.0)	38.6 (31.9-45.8)	3.8 (1.9-7.6)	9.2 (5.8-14.3)	0.5 (0.1-3.0)
Treatment in the hospital can result in complications	4.3 (2.2-8.3)	15.8 (11.2-21.7)	8.7 (5.4-13.7)	69.0 (62.0-75.3)	2.2 (0.8-5.5)
Convenience					
I would be more comfortable being treated at home than in the hospital	48.4 (41.3-55.5)	34.2 (27.8-41.4)	3.8 (1.9-7.6)	13.0 (8.9-18.7)	0.5 (0.1-3.0)
It would bother me to have nurses and doctors coming into my home^a^	1.6 (0.6-4.7)	3.8 (1.9-7.6)	0.5 (0.1-3.0)	34.8 (28.3-41.9)	59.2 (52.0-66.1)
I would like to get physical therapy visits to home	70.1 (63.1-76.3)	21.2 (15.9-27.7)	2.7 (1.2-6.2)	5.4 (3.0-9.7)	0.5 (0.1-3.0)
I would follow pain management plans	49.5 (42.3-56.6)	45.1 (38.1-52.3)	1.1 (0.3-3.9)	3.8 (1.9-7.6)	0.5 (0.1-3.0)
I would monitor medical equipment	45.7 (38.6-52.9)	41.8 (35.0-49.1)	4.3 (2.2-8.3)	7.6 (4.6-12.4)	0.5 (0.1-3.0)
I would manage medication on a schedule	52.2 (45.0-59.3)	44.0 (37.0-51.2)	1.1 (0.3-3.9)	2.7 (1.2-6.2)	NA
I would provide wound care	40.2 (33.4-47.4)	40.2 (33.4-47.4)	4.9 (2.6-9.0)	13.6 (9.4-19.3)	1.1 (0.3-3.9)
I would change the intravenous (IV) bag	28.3 (22.3-35.2)	35.3 (28.8-42.5)	4.9 (2.6-9.0)	29.3 (23.2-36.3)	2.2 (0.8-5.5)
I would change a catheter	26.6 (20.8-33.4)	29.3 (23.2-36.3)	4.3 (2.2-8.3)	38.0 (31.3-45.2)	1.6 (0.6-4.7)
I would change the feeding tubes	26.6 (20.8-33.4)	28.3 (22.3-35.2)	6.5 (3.8-11.1)	37.0 (30.3-44.1)	1.6 (0.6-4.7)
I would provide suctioning	31.5 (25.2-38.6)	33.7 (27.3-40.8)	5.4 (3.0-9.7)	27.2 (21.3-34.0)	1.6 (0.6-4.7)^c^

Abbreviation: NA, not applicable.

^a^ Negatively phrased items.

## Discussion

In this cross-sectional study, the surveyed participants generally reported feasible awareness of HH, even though agreement was not consistent over all the domains studied. Caregivers showed greater willingness to assist with basic supportive tasks; however, agreement was significantly lower for more clinically demanding responsibilities. These findings underscore important boundaries in informal caregiving capacity and suggest that caregiver participation may be task-specific rather than comprehensive. Overrating caregiver capability may lead to increased burden and compromised care quality, mainly in situations where caregivers lack formal training.

When comparing the present study findings with international research studies, differences in caregiver acceptability are more evident. According to a US study by Frasco et al,^3^ only 47.2% prefer receiving HH. Other previous study findings are consistent with the present study. They reported high satisfaction and acceptability of hospital-at-home care among caregivers and patients, with low hospitalization rates and higher willingness to provide HH care.^4,5,6^

Results should be interpreted carefully as health care system structures, caregiver expectations, and cultural norms differ substantially between settings. Another study limitation includes reliance on self-reported perceptions of caregiving, which may not reflect true burdens or performance. The study’s focus on individuals receiving hospital-at-home services could introduce selection and survivorship bias. Furthermore, the caregiving tasks evaluated were limited, excluding adverse events or safety outcomes. However, present study findings possess significant implications for policymakers and health care administrators and support Saudi Vision 2030 by enhancing cost-effective, patient-centered care.

## Notes

Supplement. Data Sharing Statement
