# Supplementary material for: Patient and Caregiver Perspectives on Hospital-at-Home in Saudi Arabia
Source: JAMA Netw Open. 2026 Mar 25;9(3):e263522. doi: 10.1001/jamanetworkopen.2026.3522 (PMC13019228; doi:10.1001/jamanetworkopen.2026.3522)
Supplement: Supplement. — Data Sharing Statement [file jamanetwopen-e263522-s001.pdf]

Data Sharing Statement

Maida. Patient and Caregivers' Perspectives on Hospital-at-Home in Saudi Arabia. *JAMA Netw Open*. Published March 25, 2026. doi:10.1001/jamanetworkopen.2026.3522

Data
Data available: Yes
Data types: Deidentified participant data
How to access data: All data are available in the manuscript
When available: With publication

Supporting Documents
Document types: None

Additional Information
Who can access the data: All the data will be available to all those who will read the article.
Types of analyses: All the analysis will be available for any purpose.
Mechanisms of data availability: The dataset will be made publicly and immediately available without restrictions upon publication.
